# Microstructure Evolution and Mechanical Properties of 0.4C-Si-Mn-Cr Steel during High Temperature Deformation

**DOI:** 10.3390/ma13010172

**Published:** 2020-01-01

**Authors:** Fei Zhang, Yang Yang, Quan Shan, Zulai Li, Jinfeng Bi, Rong Zhou

**Affiliations:** 1 School of Material Science and Engineering, Kunming University of Science and Technology, Kunming 650093, China; 15096694110@163.com (F.Z.); yangyang72199@163.com (Y.Y.); jfsd1015@126.com (J.B.); zhourong@kmust.edu.cn (R.Z.); 2 National & Local Joint Engineering Laboratory of Advanced Metal Solidification Forming and Equipment Technology, Kunming University of Science and Technology, Kunming 650093, China

**Keywords:** deformation, height-diameter ratio, pearlite, impact toughness

## Abstract

Herein, the effects of height-diameter ratios (H/D) on the microstructure evolution and mechanical properties of 0.4C-Si-Mn-Cr steel during high temperature deformation are reported. The compression experiments were performed on steel samples using Gleeble to obtain a reasonable deformation temperature, and the degree of deformation was assessed in the range of 1.5 to 2.0 H/D via forging. The forged specimens were quenched using the same heat treatment process. The hardness and impact toughness of the steel samples were tested before and after heat treatment. Grain sizes gradually increased with an increase in the compression temperature from 950 °C to 1150 °C, and the grain sizes decreased with an increase in H/D. The microstructure of the steel samples contained pearlite, bainite, martensite, and retained austenite phase. The microstructure after forging was more uniform and finer as compared to that of as-cast steel samples. The hardness and impact toughness of the steel samples were evaluated after forging; hardness first increased and then decreased with an increase in H/D, while the impact toughness continuously increased with an increase in H/D. Hence, the microstructure and properties of steel could be improved via high temperature deformation, and this was primarily related to grain refinement.

## 1. Introduction

Owing to their excellent comprehensive performance, medium carbon steels have been widely used in essential fields such as energy development, machine manufacturing, and rail traffic [1,2,3,4]. In recent years, there has been an intense focus on the improvement of material properties through deformation technologies such as hot rolling, cold rolling, and forging [5,6,7,8]. Forging is a method for refining the grain size of steel via severe plastic deformation to enhance the strength and toughness. Internal cracks and defects of raw materials can be reduced during forging, and the microstructure and grain distribution are more uniform and compact after forging [9,10].

Grain refinement during deformation has a positive effect on the microstructure and properties of steel. G.P. Chaudhari et al. [11,12,13,14] found that the grain was clearly refined after multidirectional forging of steel, which induced fine austenite grains because of continuous dynamic recrystallization and dynamic recovery. The formation of fine grains is beneficial for enhancement of tensile strength and hardness. Crystal slips are the primary mode of deformation, and the mechanism of ferrite refinement is comprised grain segmentation and recovery. Sumit et al. [15] found that when the steel was forged in pure ferrite regions, the mechanical properties of steel had the best combination of yield strength and ductility. Hot compression deformation at different temperatures (950–1150 °C) and rates (0.01–10 s^−1^) can significantly promote grain refinement of as-cast samples [16]. Yang et al. [17] conducted hot deformation experiments of 35CrMoV steel with a degree of deformation that was about 50% at temperatures of 850 °C, 950 °C, and 1050 °C and concluded that grain size increased gradually with an increase in the deformation temperature under the same strain rate. Furthermore, the performance of the forging can be further enhanced via heat treatment. Smaropoulos [18] achieved excellent tensile strength, hardness, and toughness of a low alloy steel after forging followed by heat treatment. The as-obtained medium carbon steel had extraordinary mechanical properties, and this was achieved by alternating timed quenching in water and air [19,20]. However, the current research on microstructure evolution and mechanical properties of 0.4C-Si-Mn-Cr steel under different degrees of deformation is still limited, especially in the high temperature forging process. Therefore, understanding of microstructure evolution in steels and the associated mechanism is beneficial for further formulating and improving the deformation process.

Herein, to determine an appropriate forging temperature, Gleeble simulated compression deformation of medium carbon (0.4C-Si-Mn-Cr) steel under different temperatures. Samples with different height-diameter ratios (H/D) were forged and quenched. The microstructures of specimens were characterized using optical microstructure (OM) analysis, scanning electron microscopy (SEM), X-ray diffraction (XRD), and transmission electron microscopy (TEM). The hardness and impact toughness were investigated, and the microstructure evolution and mechanical properties of 0.4C-Si-Mn-Cr steel contributed towards providing a theoretical basis for the change rule of the deformation process.

## 2. Experimental Methods

**Figure 1:**
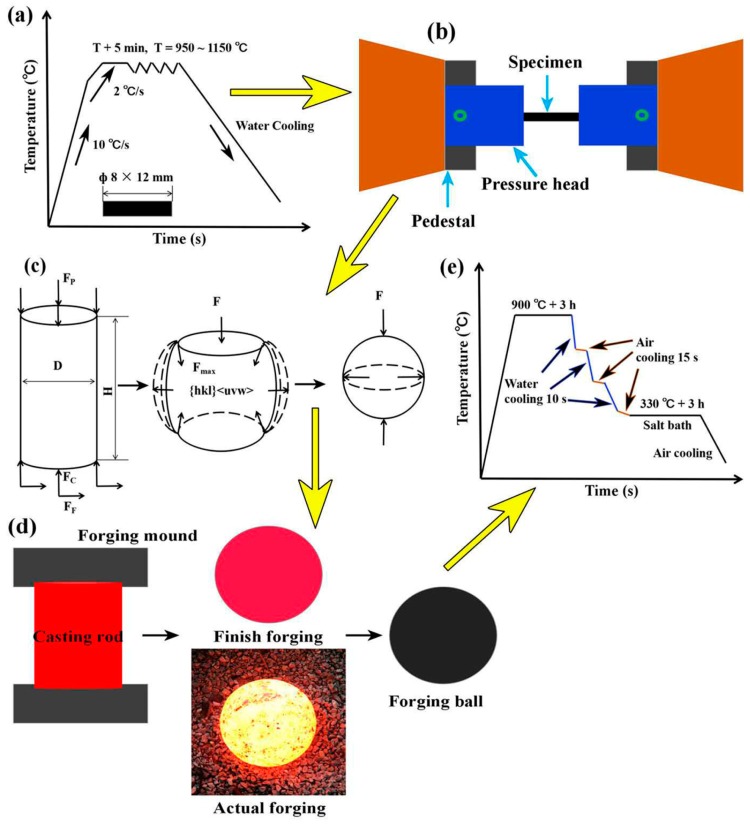
Schematic diagram of the experimental process. (**a**) High temperature compression deformation process at T = 950 °C, 1050 °C, and 1150 °C; (**b**) specimens under unidirectional compression in Gleeble; (**c**) stress and deformation of a cylinder during high temperature forging; (**d**) forging and pressing process of casting rod with height-diameter ratios (H/D) values of 1.5, 1.7, and 2.0; (**e**) heat treatment process of casting and forging.

The chemical composition of the steel investigated in this work is listed in Table 1. The steel was smelted and poured into a cylindrical billet in an intermediate frequency furnace. A direct reading spectrometer was used to determine the chemical composition of the tested alloy. A schematic diagram of the experimental process is provided in Figure 1. The billet was processed into a round rod of size Φ8 × 12 mm. Specimens were heated to 50 °C below the predetermined temperature at a rate of 10 °C/s on a Gleeble-1500D dynamic thermal simulator (Data Sciences International INC, St. Paul, MI, USA), and then, the heating rate was reduced to 2 °C/s. The strain rate was 5 s^−1^, and deformation was 50%. Compression tests were conducted at temperatures of 950 °C, 1050 °C, and 1150 °C and maintained at the specified temperatures for 5 min followed by water quenching to cool the sample to room temperature (Figure 1a,b). The round bar was subjected to multidirectional force and then formed into a ball in a high temperature forging process (Figure 1c). Cylindrical billets with different H/D values (1.5, 1.7, and 2.0) were taken to 1050 °C for hot forging, and the billets were forged into a sphere of 150 mm (Figure 1d). The heat treatment was carried out by alternating the cycle cooling process in water and air (Figure 1e). After forging, samples were subjected to a quenching treatment under the same conditions. Moreover, all the samples were heated to 900 °C for 3 h, followed by alternating cycle cooling in water and air (after water cooling, the sample was cooled in air). Because the cooling rate of steel in water was higher than that in air, the temperature of samples could be reduced relatively uniformly to the isothermal region. As the quenching temperature decreased, the slope of the curve during water cooling was reduced gradually. The time of water cooling was 10 s, and that of air cooling was 15 s. The samples were treated at 330 °C for 3 h in a salt bath and further cooled to room temperature in air.

The specimens were prepared at half the distance from the center of the instrument before and after heat treatment. The samples were then processed into unnotched impact specimens with dimensions of 10 × 10 × 55 mm. Hardness and impact tests were performed using a Rockwell tester (HR-150A, Dongshan, China) and semi-automatic tester (JB-300B, Dongshan, China). The specimens used a mixture of saturated picric acid, purified water, detergent, and hydrochloric acid to corrode the grains. The diameter of the grain in different fields was measured using the cut line method in the experiment, and then, the average value was counted as the grain size. The samples were corroded using 4% nitric acid alcohol solution and picric acid solution. XRD (MiniFlex 600, Rigaku, Japan) was performed on a diffractometer (Copper Kα radiation) at 10°/min for the counting time from 30° to 100°. Furthermore, for TEM, the specimens were prepared via electrolytic double spraying of 5% perchloric acid and 95% alcohol solution. The microstructure of the steel was observed using an optical microscope (Nikon ECLIPSE MA200, Japan), scanning electron microscope (ZEISS EVO18, Baden-Wurttemberg, Germany), and transmission electron microscope (FEI G220, Hillsboro, Oregon State, U.S.).

## 3. Results

**Figure 2:**
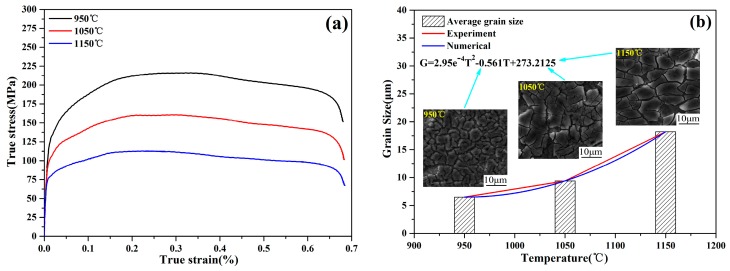
Stress–strain curves (**a**) and grain size data (**b**) of compressed 0.4C-Si-Mn-Cr steel under temperatures from 950 to 1150 °C.

Stress–strain curves and the grain size of the compressed steels at various temperatures are shown in Figure 2. The shape of the flow curve was mainly unimodal. At different temperatures, with an increase in strain, the true stress first increased to a maximum value and then decreased to the steady-state region. When the temperature increased from 950 to 1150 °C, the maximum flow stress decreased from 219.93 to 117.23 MPa. This may be because of the increase in temperature and the activation energy of thermal deformation. Furthermore, the kinetic energy of atoms increased, and the critical shear stress slip line decreased. The behavior of strain softening was considered to be due to new dynamic recrystallization (DRX), and the asymptotic behavior was characteristic of dynamic recovery (DRV) [21,22]. The shape of the grains was a polygon or a block. With an increase in compression temperature, the grain size increased significantly. The maximum value grain size was obtained under a compression temperature of 1150 °C. When the compression temperature was more than 1050 °C, the microstructure appeared to be clearly the feature of an equiaxed crystal. DRX incompletely produced granular crystal grains at a low temperature. Moreover, the average grain size gradually increased from 6.2 to 17.8 μm with an increase in the compression temperature. The variation rule of the actual grain size was obtained via numerical fitting. The relationship between grain size and compression temperature is as follows:

(1) $$G=2.95e^{-4}T^{2}-0.561T+273.2125$$

where *G* is the grain size (μm) and *T* is the compression temperature (°C).

**Figure 3:**
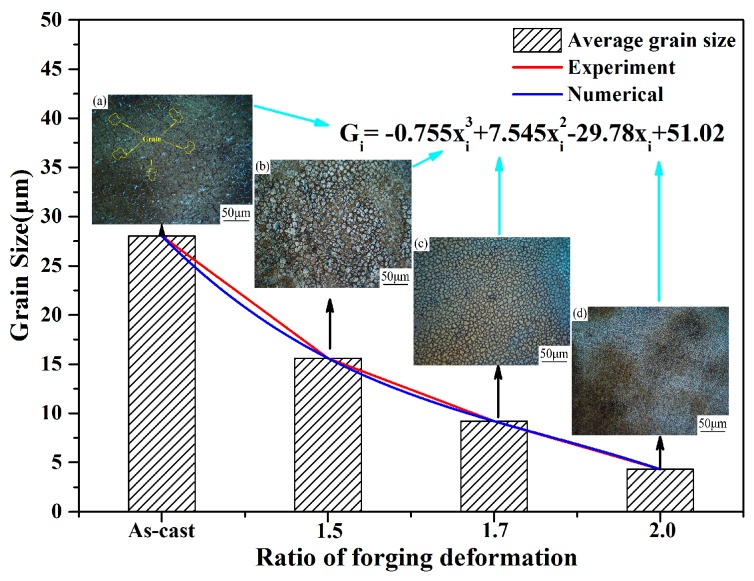
Grain size of 0.4C-Si-Mn-Cr steel with different H/D values for the deformation process. (**a**) As-cast, (**b**) H/D = 1.5, (**c**) H/D = 1.7, and (**d**) H/D = 2.0.

The grain sizes were fine for the compression temperatures in the range of 950–1050 °C. Therefore, to obtain fine original austenite grains before heat treatment, the forging temperature was selected to be 1050 °C, which is in the range of 950–1050 °C. The sizes of grains with different H/D values are provided in Figure 3. The results showed that the shape of the samples was elliptical and band-like, and the grain size decreased with an increase in the H/D value. For cast steel, the grain size was about 26.4 μm, and the grains were shaped as irregular blocks. Furthermore, the distribution was relatively dispersed, and the grain roundness was not good enough. When the H/D value was from 1.5 to 2.0, the grains were mainly elliptical. After forging, the grain size decreased to 3.6 μm, and the distribution of grains was relatively uniform and dense. With forging, the variations in grain size with different H/D values were obtained via numerical fitting:

(2) $$G_{i}=-0.755x_{i}^{3}+7.545x_{i}^{2}-29.78x_{i}+51.02\left(i\in R,i\neq 0\right)$$

where *G_i_* is the grain size (μm) and *x_i_* is the height-diameter ratio (H/D).

**Figure 4:**
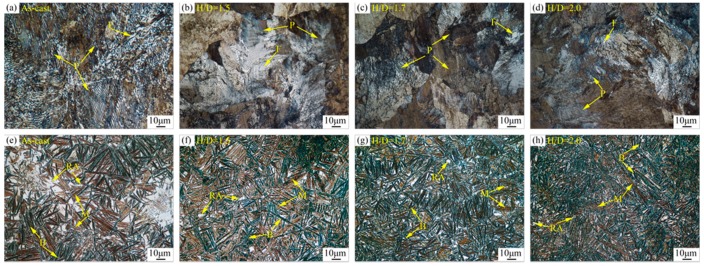
Optical microstructure (OM) of 0.4C-Si-Mn-Cr steel before and after heat treatment with different H/D values for the deformation process. (**a**–**d**) Before heat treatment and (**e**–**h**) after heat treatment. P: pearlite, F: ferrite, B: bainite, M: martensite, and RA: retained austenite.

The optical microstructure (OM) of 0.4C-Si-Mn-Cr steel before and after heat treatment with different H/D values is shown in Figure 4. The microstructure was primarily composed of pearlite and ferrite before the heat treatment, and the pearlite and ferrite were distributed among each grain. As shown in Figure 4a–d, the size of the pearlite in the forged samples was finer than that of the pearlite in the as-cast steel. The pearlite in the forged steel was mainly distributed on the shear bands, and the pearlite was deformed in the growth direction. Compared with forged steels, the as-cast specimen had a distinct grain orientation, which could be because a temperature gradient field was formed in the crystal body due to the quantitative loss of heat in the uncompressed state. This led to the formation of fibrous tissue parallel to the direction of heat dissipation. The microstructures of samples after heat treatment for different H/D values are shown in Figure 4e–h. The microstructure was mainly composed of acicular bainite, martensite, and retained austenite. Compared with Figure 4e, the bainite and martensite of specimens were finer and had a dispersed distribution, as shown in Figure 4f–h. According to the color metallographic techniques [19], with an increase in H/D, the bainite content increased gradually while the martensite content decreased.

**Figure 5:**
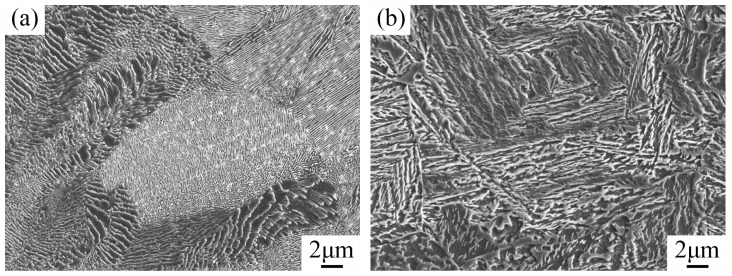
SEM micrographs of the microstructure in the tested steel. (**a**) Before heat treatment and (**b**) after heat treatment.

**Figure 6:**
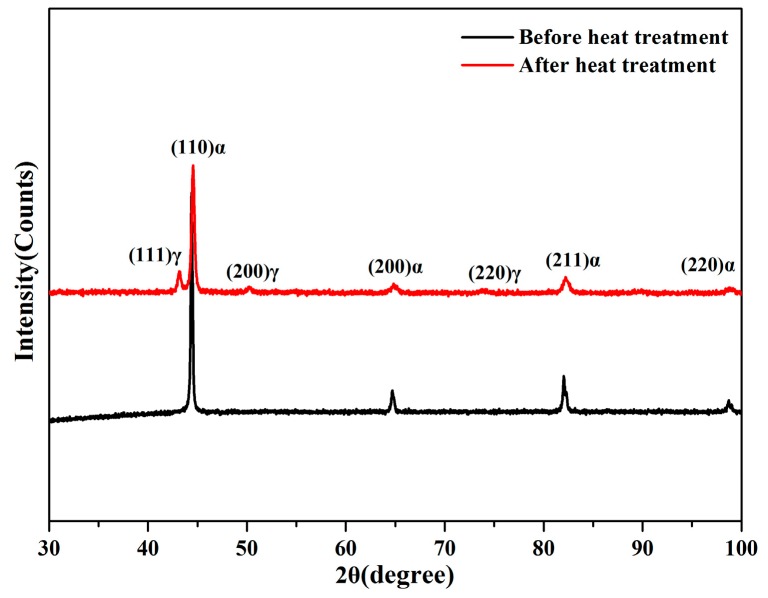
XRD spectra of the tested steel treated under different conditions.

Figure 5 shows SEM images of steel before and after heat treatment. Figure 5a is an SEM image of the sample with an H/D value of 2.0 before heat treatment. Different pearlite clusters were distributed and grew along the grain boundary. The sizes of ferrite and cementite in all the grains were different and regularly arranged in a specific direction. The SEM microstructure of steel after heat treatment is shown in Figure 5b, and the microstructure was similar to that shown in Figure 4e–h. Figure 6 shows the XRD spectra for the steel samples before and after heat treatment, and the peaks observed before and after heat treatment corresponded to the body-centered cubic structure. These results showed that the sample had the face centered cubic structure after heat treatment. With the combined analysis of Figure 4 and Figure 5, it could be confirmed that before treatment, the body centered cubic structure of the steel was ferrite, while after treatment, there was a mixed structure of bainite and martensite. The face centered cubic structure was the retained austenite phase after quenching, and these results were consistent with the experimental results.

**Figure 7:**
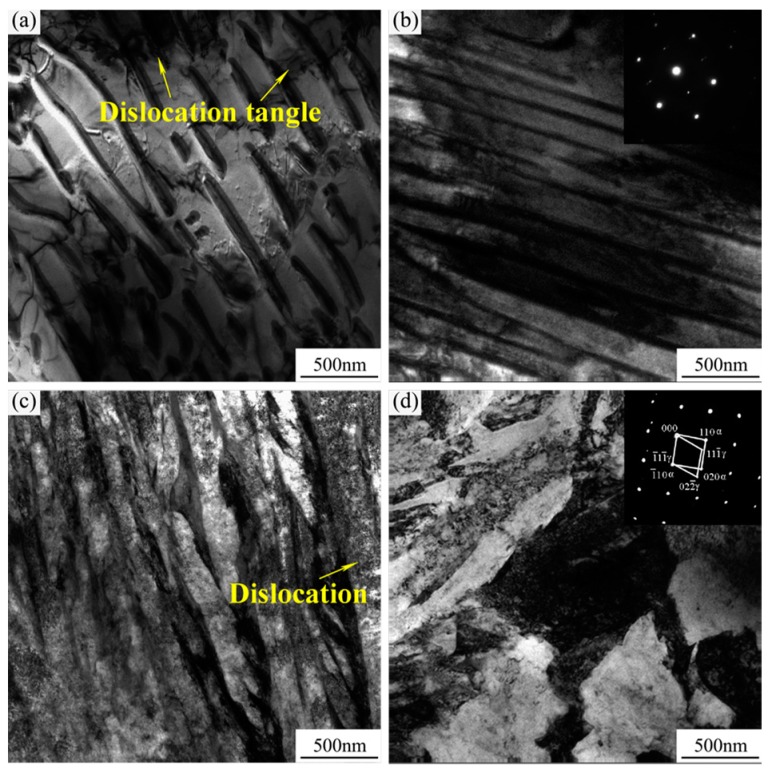
TEM images of the morphology and SAED of steel with an H/D value of 2.0 in the deformation process. (**a**–**b**) Before heat treatment and (**c**–**d**) after heat treatment.

Figure 7 shows TEM images and the selected area electron diffraction (SAED) pattern of steel with an H/D value of 2.0. As shown in Figure 7a, after forging, the steel had a large number of dislocation lines and dislocation tangles between the ferrite and cementite. Furthermore, cementite (<100 nm) is observed in Figure 7b. The deformation ability of pearlite with ferrite and cementite was variable because of different stresses during high temperature forging. A dislocation in the ferrite formed because of the plastic deformation of the pearlite during forging. When there was a large amount of deformation, the layer size and grain size of pearlite were fine. The deformation resistance decreased via adjustments in the orientation of lamella during continuous impact compression. The cementite in the pearlite grain group gradually fractured, and the ferrite formed a high density of dislocations and a substructure that could reduce the strain energy [23]. TEM images of the morphology of the steel after heat treatment with an H/D value of 2.0 are shown in Figure 7c–d. After heat treatment, the steel obtained the multiphase microstructure that included dislocation-type lath martensite, bainite, and retained austenite and a number of dislocations gathered around the martensite. According to the inserted SAEDP in Figure 7d, the orientation relationships of the multiphase microstructure were ($\bar{1}$10)α // ($\bar{1}$1$\bar{1}$)γ. In addition, the retained austenite was mainly found to be film-like and blocks.

**Figure 8:**
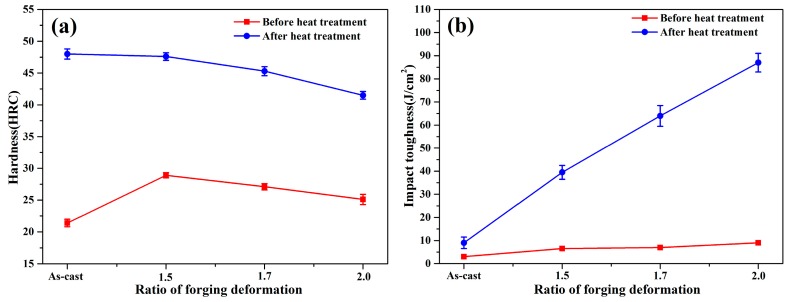
Mechanical properties of the 0.4C-Si-Mn-Cr steel showing the development of (**a**) hardness and (**b**) impact toughness before and after heat treatment.

The mechanical properties of steel with different H/D values are shown in Figure 8. As shown in Figure 8a, the hardness values (40–45 HRC) of the steel samples after heat treatment were larger than the hardness values (21–29 HRC) of the steel samples before heat treatment. As shown in Figure 8b, the impact toughness after heat treatment was greater than 12 J/cm^2^, which was higher than that of the steel samples before heat treatment (3–9 J/cm^2^). The hardness and the impact toughness were significantly enhanced after heat treatment. Before heat treatment, with an increase in the H/D value, hardness first increased and then decreased. However, the impact toughness of the samples before and after heat treatment increased linearly.

## 4. Discussion

**Figure 9:**
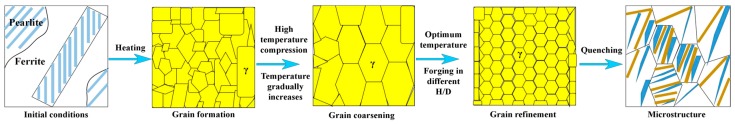
Microstructure evolution mechanism of medium carbon 0.4C-Si-Mn-Cr steel during the process of compression-forging-heat treatment.

Figure 9 presents a series of schematic diagrams of the microstructure evolution of steel during the process of compression-forging-heat treatment. When the temperature was greater than point *A*_1_, pearlite and ferrite dissolved and formed austenite grains. When the specimens began to yield, plastic strain and recrystallization driving force increased with an increase in compression temperature. The grain boundary migration rate accelerated, and this promoted rapid grain growth.

According to the kinetics of grain growth [24]:

(3) $$G_{t}^{m}-G_{t_{0}}^{m}=k\left(t-t_{0}\right)$$

where *Gt*_0_ and *G_t_* are the grain diameter (μm) at the times *t*_0_ and *t*, respectively. *m* is the average mobility of the grain boundary, and *m* depends on the index of *G_t_/G_t0_*. *k = k_0_exp(−Q/RT)*, where *Q* is the activation energy of the grain boundary and *T* is the heating temperature.

According to Equation (3), when time is constant, *m* is proportional to *exp(−Q/RT)*; thus, the rate of grain growth is related to *m*. When the temperature is higher, the grain boundary migration and the growth rate were faster due to the increased atomic diffusion rate; also, the grain size increased exponentially with an increase in temperature. Temperature affects the plastic deformation of a material, which as a consequence, directly influences the microstructure evolution. The growth rate of grains at a low temperature was lower than that at a high temperature, which was because recovery and recrystallization were more pronounced at a high temperature.

The diameter of the cross-section of the round bar decreased during forging. Under the action of external force, the round bar was deformed and expanded outward in the radial direction. The two end faces were elongated along the vertical axis (Figure 1c). The dislocation density of pearlite during the forging deformation process increased, and its value changed from 2.02 × 10^14^ m^−2^ to 6.07 × 10^14^ m^−2^; this provided nucleation sites for cementite and promoted diffusion of iron and carbon atoms. An increase in the H/D value accelerated the migration of atoms along the dislocation line, and this led to a decrease in the deformation resistance. When the strain was higher than the critical strain, the lattice was distorted because the number of slippages and twinning increased via atoms through dislocation. The initial grains began to break, and the slip resistance increased in this case. Dislocation density and deformation energy were formed in the ferrite, and this was beneficial for obtaining fine grains during DRX [25,26]. Furthermore, the temperature of the core slowly decreased because of the large volume of steel, providing sufficient time for grain recovery in the forging process. However, with a continuous decrease in temperature during the forging process, it was difficult to recrystallize deformed austenite because delayed nucleation inhibited DRX [27]. In addition, the high content of Si increased the hot deformation energy and reduced the rate of DRX, which made the steel deformation difficult, and the grain growth gradually stopped [28,29,30]. The microstructure of the undeformed billet was relatively stable under same heating conditions and still retained a large grain size. When the billet was air cooled to room temperature after forging, transformation of the ferrite and pearlite occurred during the cooling process [31]. Because of nucleation and grain growth during the transformation process, it was difficult to obtain quantitative information for DRX during deformation.

The hardness and impact toughness of the steel sample obviously increased under the same quenching conditions. The grain size decreased after forging, and microstructure refinement improved the hardness and toughness of the metal [32]. After heat treatment, the pearlite of the forged state transformed into bainite, martensite, and retained austenite. The increase in the martensite content was beneficial for enhancing the hardness, and bainite and film-like retained austenite clearly improved the impact toughness [33]. The transformation induced plasticity (TRIP) effects resulted from the deformation during forging, and this contributed to the enhanced toughness because the austenite was rich in manganese and carbon [34]. Smaller prior austenite grains formed a finer martensite lath after heat treatment, whereas the longer martensite in the specimens also meant that there were larger grains before austenite transformed to bainite and martensite [35,36]. With an increase in deformation, the hardness of the forged billet slightly decreased before and after heat treatment. The work hardening effect was inherited by the subsequent microstructural transformation process due to thermal deformation. The material appeared to undergo dynamic recovery and flow softening because of the influence of the annihilation of dislocation when the strain was further increased to some extent [37,38]. With increased deformation at different values of H/D, the specimens were completely recrystallized, and this reduced the deformation energy storage and led to decreased hardness. However, the hardness of steel was improved compared to that of the as-cast steel because of deformation. 

## 5. Conclusions

(1) With an increase in deformation temperature in the range of 950–1150 °C, the grain size of samples increased from 6.2 μm to 17.8 μm. The relationship between the grain size and temperature conformed to the fitting formula (Equation (1)). The steel had an abundance of blocky grains during the deformation process. Moreover, the grain size decreased from 26.4 μm to 3.6 μm with an increase in the H/D value. The relationship between the grain size and H/D was close to Equation (2).
(2) The microstructure was pearlite, ferrite, bainite, martensite, and retained austenite with different H/D values. The microstructure was clearly more uniform and finer than that of the as-cast steel. The optimum deformation process was a heating temperature that was less than 1050 °C, and the H/D value was 2.0. Changes in grain size depended on the grain nucleation and dislocation movement during forging.
(3) Compared with steel that was not subjected to heat treatment, the hardness and impact toughness of the steel samples after heat treatment showed obvious improvements. When the H/D value was 2.0, the maximum impact toughness was 87 J/cm^2^. Heat treatment had a significant contribution to improving the mechanical properties of the forged samples. Hardness decreased slightly with an increase in the H/D value. Furthermore, the impact toughness gradually increased, and this was related to the microstructure, dynamic recovery, and softening of the grains.

## Figures and Tables

**Table 1:** Chemical composition of the 0.4C-Si-Mn-Cr steel.

Element	C	Si	Mn	Cr	Fe
Content (wt.%)	0.37–0.60	1.60–2.0	2.0–2.5	0.55–0.85	Balance
